# Supplementary material for: Predicting cell-to-cell communication networks using NATMI
Source: Nat Commun. 2020 Oct 6;11:5011. doi: 10.1038/s41467-020-18873-z (PMC7538930; doi:10.1038/s41467-020-18873-z)
Supplement: Supplementary file 1 — Supplementary Information [file 41467_2020_18873_MOESM1_ESM.docx]

**Supplementary information**

Predicting cell-to-cell communication networks using NATMI

Hou et al.

**Supplementary Figures:**

**Supplementary Fig. 1: Overview of cell-to-cell communication analysis of scRNA-seq data using NATMI.** **a**) Inputs for NATMI consist of user provided (**i**) single-cell gene expression data with normalized expression values for each cell and a mapping file between each single cell and cell type, and (**ii**) a curated ligand-receptor pair list (connectomeDB2020 is provided in NATMI) or a user-defined ligand-receptor pair list. These are used to extract the expression profiles of each **b**) ligand and **c**) receptor in each cell type. From this information NATMI allows users to **d**) examine the cell types communicating using selected ligand and receptor pair, **e**) examine ligands and receptors used for communication between selected pair of cell types and **f**) summarize cell-to-cell communication between all cell types in the input data. **g**) NATMI also allows the user to compare two networks to identify differences.

**Supplementary Fig. 2: Complexity of the ligand-receptor-mediated multicellular interaction network.** Nodes represent cell types, simple edges and hyperedges represent ligand-receptor-mediated communication, and the thickness of an edge scales with the edge weight. **a)** Simple representation of communication between a pair of cells based on a single ligand-receptor pair. **b)** Communication between a pair of cells based on multiple ligand-receptor pairs expressed at various levels. **c)** Communication between multiple cell types based on multiple ligand-receptor pairs. As each ligand and receptor (making up a receptor-ligand pair) can be expressed in different cell types at different levels, edges are shown as directed weighted hyperedges. **d)** In NATMI we reduce **c** to a set of weighted directed simple edges. We refer to these as cell-connectivity summary edges.

**Supplementary Fig. 3: Three different edge filtering strategies.** Here we use a theoretical network consisting of three cell types and nine different ligand-receptor pairs (coloured edges) to demonstrate different filtering strategies. Each ligand-receptor pair has different expression levels and specificity. Nodes represent cell types, edges represent ligand-receptor-mediated communications, and the thickness of an edge shows the expression weight of the edge. **a)** Original unfiltered cell-to-cell communication network. **b)** Edges in **a** are filtered to only keep edges between the cell expressing the highest level of the ligand and the highest level of the receptor (L_max_-R_max_). **c)** Edges in **a** are filtered based on a specificity weight. **d)** Edges in **a** are filtered based on a minimum expression weight. Under **a-d** we show the resulting cell-connectivity summary networks based on simple edge count, total expression or total specificity.

**Supplementary Fig. 4: Whole-body signalling distribution analyses based on secreted and plasma-membrane ligands in Tabula Muris. a**) The number of ligands (x-axis) versus numbers of receptors (y-axis) detected in each cell type. Each data-point corresponds to a cell type, colors indicate broad lineage classes and the detection rate threshold of 20% was used. The red lines show the mean numbers of ligands and receptor. **b**) The number of ligand-receptor pairs co-detected in at least one cell type (at the expression level of 0CPM and 10CPM) as a function of the proportion of single-cells co-expressing the L-R pair. In panels **c-j** we repeat the analysis shown in main **Figure 4c-f** but instead either use simple edge-counts (**c-f**) or summed expression (**g-j**) to rank cell connectivity summary edges.

**Supplementary Fig. 5: Heatmaps showing whole-body signalling network analyses based on secreted and plasma-membrane ligands in the Tabula Muris single-cell dataset.** Each element in **a**&**b** corresponds to the total specificity weight of a cell-connectivity-summary edge from cell type A to cell type B, color bars in the middle indicate broad lineage classes. A detection rate threshold of 20% was used for **a** and **b.** **a**) The heatmap of cell-connectivity-summary edges in Tabula Muris based on communication via secreted ligands. **b**) The heatmap of cell-connectivity-summary edges in Tabula Muris based on communication via plasma-membrane ligands.

**Supplementary Fig. 6: Communities and autocrine signalling inferred running NATMI on the FANTOM5 dataset.** Visualization of cell types connected by the top ten summed-specificity edges in the FANTOM5 human primary cell data based on **a**) secreted ligands and **b**) plasma-membrane ligands. Coloured shadows highlight the isolated communities found in each network, edge labels correspond to the total-specificity weight of the edge. Dashed oval in **a** highlights predicted broadcasting from hepatocytes to multiple cell types via secreted ligands. Dashed in oval in **b** highlights a set of two brain-derived cell types communicating via plasma membrane-based ligands. Panels **c-f**, show a similar analysis as used in **Fig. 4c-f**, however in this case only autocrine edges are shown. **c**) Shows the distribution of ranks of secreted ligand mediated outgoing edges, **d**) Shows the distribution of ranks of secreted ligand mediated incoming edges, **e**) Shows the distribution of ranks of plasma-membrane ligand mediated outgoing edges, **f**) Shows the distribution of ranks of plasma-membrane ligand mediated incoming edges.

**Supplementary Fig. 7: Runtime comparison of NATMI and CellPhoneDB on various vCPUs.** The x-axis is the number of vCPUs used to run both tools; the y-axis is the average time in seconds each tool takes to build the cell-to-cell communication network in the test dataset using connectomeDB2020.

**Supplementary Fig. 8: Runtime of extracting edges from benchmark networks on various vCPUs with NATMI.** The x-axis is the number of vCPUs used to run NATMI; the y-axis is the average time in seconds NATMI took to extract all edges from the ten benchmarking networks using connectomeDB2020.

**Supplementary Notes**

With the size and complexity of single cell expression profiles, it is critical to build cell-to-cell communication networks reasonably fast while providing users with reliable estimates on the edge weighting in a given dataset. Here, we compare our Network Analysis Tool (NATMI) to CellPhoneDB v2.0 [1] (the only analysis tool currently available for intercellular communication network inference) using connectomeDB2020 ligand-receptor interaction database and an example single-cell dataset from CellPhoneDB. We first evaluate their runtime performance (**Supplementary Note 1**) and then compare the predicted edges (**Supplementary Note 2**). Besides the differences in the execution times (NATMI generally outperforms CellPhoneDB, especially when using limited computational resources), we find that under the identical criteria NATMI and CellPhoneDB predict the same potential edges, yet the two software differ in assigning weights to a given edge. These differences, in turn, might affect the final specific edge identification (as further described).

**Supplementary Note 1: runtime comparison between NATMI and CellPhoneDB**

We evaluated runtime performance for NATMI and CellPhoneDB v2.0 (the latest version as of March 2020), on a virtual machine equipped with 16 vCPUs (using connectomeDB2020 interaction database and an example single-cell dataset from CellPhoneDB with ten cells from four cell types). Both NATMI and CellPhoneDB were tested with default settings and with no threshold on ligand receptor detection (NATMI by default predicts all potential edges in the dataset while CellPhoneDB only uses ligands and receptors above a given detection threshold, which for this comparison was set to zero.) Moreover, a recently introduced subsampling function of CellPhoneDB, which uses geometric sketching [2] and generally results in only a mild reduction of the computing time, was not tested as the inferred edges could potentially be unreliable for the small data set used here.

We then tested each software by running it with 1, 2, 4, 6, 8, 10, 12, 14, and 16 vCPUs and repeating each run 10 times (**Supplementary Fig. 7**). When using a single vCPU, CellPhoneDB took ~5.5 times longer on average to complete than NATMI (483.6s vs 87.2s), which can mainly be attributed to elaborate statistical inference procedure used by CellPhoneDB. The running times of CellPhoneDB, however, were noticably reduced when using multiple vCPUs (483.6s for one vCPU, 256.2s for two vCPUs, 140.5s for four vCPUs, 85.5s for eight vCPUs). For the dataset tested here, NATMI was only able to take a slight advantage of multithreading (the average runtime shortened from 87.4s to 73.9s when using four vCPUs as compared to one vCPUs). Nevertheless, running CellPhoneDB with 8 vCPUs only slightly outperformed NATMI with a single vCPU taking 85.5 sec vs 87.4 sec, respectively.

Although NATMI supports multi-core computing, **Supplementary Fig. 7** shows that NATMI performed best using four vCPUs. We thus next examined the performance of NATMI simulating various computational demands using our virtual machine. Since the size of a communication network derived from a dataset is roughly proportional to the number of cell-types/clusters in the dataset and the example dataset has ten individual cells, we subsampled the data and generated networks of different cluster sizes: 10 clusters (3309 edges), 9 clusters (2837 edges), 8 clusters (2331 edges), 7 clusters (1760 edges), 6 clusters (1319 edges), 5 clusters (1075 edges), 4 clusters (844 edges), 3 clusters (516 edges), 2 clusters (418 edges) and 1 cluster (188 edges). NATMI was then tested on these 10 networks with 1, 2, 4, 6, 8, 10, 12, 14, and 16 vCPUs, respectively. The execution time averaged over 10 repeated runs for each network is shown in **Supplementary Fig. 8**. For networks with larger cluster numbers, NATMI took more advantage of multithreading. For example, the execution time for the network with 10 clusters had decreased by 45.3% when using 10 cores as compared to using a single core (84.1s vs. 153.8s). For a comparison, we observed only a 20.2% decrease in execution time for the network with 5 clusters (76.9s vs. 96.4s) and essentially no such difference was observed for the network with 1 cluster (73.9s vs. 75.0s).

Taken together, this shows that NATMI benefits from parallel computing, especially for datasets with bigger cell-type coverage. In addition, regardless of the size of a dataset used here, NATMI took less time to predict all edges using a single vCPU than CellPhoneDB with 16 vCPUs making it particularly practical to use on platforms with limited computational resources (laptops, PCs).

**Supplementary Note 2: edge comparison between NATMI and CellPhoneDB**

In addition to efficient execution, the ability to identify biologically meaningful edges is an important aspect for intercellular network prediction tools. We thus examined the output of CellPhoneDB and NATMI on the example single-cell dataset tested in Supplementary Note 1 (10 cells, 4 cell types) at the detection rate threshold of 20%. Using this criterion, both CellPhoneDB and NATMI predicted 938 identical edges (see **Supplementary Data 10**). Although both software utilize the average expression levels of ligands and receptors, the ‘*edge weights’* obtained are different as CellPhoneDB uses mean while NATMI uses the product of the average expression levels of the ligand and its cognate receptor. From **Supplementary Data 10**, if the difference between the average expression levels of the ligand and its cognate receptor were huge, the ranks of edges based on these two inferred edge weights would be drastically different. Nevertheless, edge weights are only used to determine the specificities of the edges via the same ligand-receptor pair.

Since housekeeping edges that connect all cell types are likely to be selectively neutral compared to specific ones used by a few cell types, both CellPhoneDB and NATMI use their own ‘*edge specificity’* calculation methods to prioritize the ligand-receptor-mediated interactions. We therefore compared the specificity of each edge from CellPhoneDB and NATMI derived from the average expression levels of ligands and receptors, as follows. CellPhoneDB permutes cell labels 1,000 times to generate the null distribution of expression levels of ligands and receptors in each cell type and then, by comparing the actual expression levels with the simulated values, infers the likelihood of a receptor–ligand complex specifically used by a cell-type pair. NATMI, on the other hand, calculates the specificity of the ligand/receptor in the cell type as the proportion of a ligand/receptor’s expression level in a cell type to the total expression levels of the ligand/receptor in the dataset and then multiplies these specificities to obtain the specificity of the corresponding edge.

By default, CellPhoneDB shows all edges for the same ligand-receptor pair with significant P-values (less than 0.05) and then the user needs to manually select the biologically relevant edges. In **Supplementary Data 10**, the P-values of two edges using ANOS1-SDC2 are both less than 0.05, while the NATMI specificity of the edge from Myeloid to itself is 0.83. NATMI's specificity measurement thus automatically identified the dominant edge without user intervention. On the other hand, there are 16 edges via B2M-HLA-F. The weights of these edges generated by CellPhoneDB are similar but one edge has a significant P-value (0.044) and the third biggest edge weight. On the contrary, the weights of these edges generated by NATMI are highly variable, but the corresponding specificities are all less than 0.1.

Overall, 64 edges are only specific for CellPhoneDB (P-value ≤ 0.05), 117 edges are only specific for NATMI (specificity > 0.2), and 215 edges are specific for both (see **Supplementary Data 10**). Considering that a housekeeping ligand-receptor pair could connect 16 cell-type pairs in the dataset, for those ligand-receptor pairs who have at most 4 edges (specificity>0.2), NATMI is more likely to recognize them as significantly specific.

In conclusion, NATMI is an efficient (fast and using limited computational resources) cell-cell network analysis tool that extracts relevant and generally specific cell-cell interaction edges without requiring user intervention.

**Supplementary References**

1. Efremova, M., et al., *CellPhoneDB: inferring cell–cell communication from combined expression of multi-subunit ligand–receptor complexes.* Nature Protocols, 2020: p. 1-23.

2. Hie, B., et al., *Geometric sketching compactly summarizes the single-cell transcriptomic landscape.* Cell systems, 2019. **8**(6): p. 483-493. e7.
