# Supplementary material for: Predicting cell-to-cell communication networks using NATMI
Source: Nat Commun. 2020 Oct 6;11:5011. doi: 10.1038/s41467-020-18873-z (PMC7538930; doi:10.1038/s41467-020-18873-z)
Supplement: Supplementary file 3 — Description of Additional Supplementary Files [file 41467_2020_18873_MOESM3_ESM.pdf]

Description of Additional Supplementary Files

Supplementary Data 1: ConnectomeDB2020 ligand-receptor pair list

Supplementary Data 2: The flat output file of ligand-receptor-mediated communication edges between cluster/cell-types in Skelly *et al.* [11] dataset.

Supplementary Data 3: The ligand-receptor pairs co-detected in each cell type of Tabula Muris dataset at the detection rate threshold of 20%.

Supplementary Data 4: Potential for self-signalling predicted in single cells from the Tabula Muris dataset.

Supplementary Data 5: The adjacency matrices of cell-connectivity-summary networks from the Tabula Muris dataset weighted by the summed-specificity between each of the 117 cell-types

Supplementary Data 6: 15 most specific ligand-receptor pairs involved in the top ten summed-specificity edges in Fig. 5

Supplementary Data 7: The related adjacency matrices for the delta networks shown in Fig. 6.

Supplementary Data 8: All edges detected in the 3-month-old and/or 18-month-old murine mammary gland.

Supplementary Data 9: Comparison of currently available network analysis tools for single-cell gene expression data.

Supplementary Data 10: Extracted edges in the example dataset using CellPhoneDB and NATMI with the detection rate threshold set to 20% and ConnectomeDB2020 used as the ligand-receptor database.
