# Supplementary material for: Predicting cell-to-cell communication networks using NATMI
Source: Nat Commun. 2020 Oct 6;11:5011. doi: 10.1038/s41467-020-18873-z (PMC7538930; doi:10.1038/s41467-020-18873-z)
Supplement: Supplementary file 14 — Reporting Summary [file 41467_2020_18873_MOESM14_ESM.pdf]

nature research

Corresponding author(s): Alistair R. R. Forrest

Last updated by author(s): Sep 2, 2020

Reporting Summary

Nature Research wishes to improve the reproducibility of the work that we publish. This form provides structure for consistency and transparency in reporting. For further information on Nature Research policies, see our Editorial Policies and the Editorial Policy Checklist.

Statistics

For all statistical analyses, confirm that the following items are present in the figure legend, table legend, main text, or Methods section.

n/a	Confirmed	
☒	☐	The exact sample size (*n*) for each experimental group/condition, given as a discrete number and unit of measurement
☒	☐	A statement on whether measurements were taken from distinct samples or whether the same sample was measured repeatedly
☒	☐	The statistical test(s) used AND whether they are one- or two-sided<br>*Only common tests should be described solely by name; describe more complex techniques in the Methods section.*
☒	☐	A description of all covariates tested
☒	☐	A description of any assumptions or corrections, such as tests of normality and adjustment for multiple comparisons
☒	☐	A full description of the statistical parameters including central tendency (e.g. means) or other basic estimates (e.g. regression coefficient) AND variation (e.g. standard deviation) or associated estimates of uncertainty (e.g. confidence intervals)
☒	☐	For null hypothesis testing, the test statistic (e.g. *F*, *t*, *r*) with confidence intervals, effect sizes, degrees of freedom and *P* value noted<br>*Give P values as exact values whenever suitable.*
☒	☐	For Bayesian analysis, information on the choice of priors and Markov chain Monte Carlo settings
☒	☐	For hierarchical and complex designs, identification of the appropriate level for tests and full reporting of outcomes
☒	☐	Estimates of effect sizes (e.g. Cohen's *d*, Pearson's *r*), indicating how they were calculated

Our web collection on statistics for biologists contains articles on many of the points above.

Software and code

Policy information about availability of computer code

Data collection	All data analyzed within this manuscript are publicly available. No additional software was used for the data collection process.
Data analysis	This manusript presents a new Python package NATMI, which are built upon Python 3.7.6. NATMI have many dependencies, including pandas (v1.0.3), XlsxWriter (v1.2.8), xlrd (v1.2.0), seaborn (v0.10.1), igraph (v0.7.1), NetworkX (v2.4), PyGraphviz (v1.5), bokeh (v2.0.2) and holoviews (v1.13.2). The algorithms of NATMI are described in the methods, and its implementation in Python is available on GitHub (https://github.com/forrest-lab/NATMI).

For manuscripts utilizing custom algorithms or software that are central to the research but not yet described in published literature, software must be made available to editors and reviewers. We strongly encourage code deposition in a community repository (e.g. GitHub). See the Nature Research guidelines for submitting code & software for further information.

Data

Policy information about availability of data

All manuscripts must include a data availability statement. This statement should provide the following information, where applicable:

- Accession codes, unique identifiers, or web links for publicly available datasets
- A list of figures that have associated raw data
- A description of any restrictions on data availability

The FANTOM5 CAGE expression data for human protein-coding genes in the 144 human primary cells are publicly available on FANTOM5 website (https://fantom.gsc.riken.jp/5/suppl/Ramilowski_et_al_2015/data/ExpressionGenes.txt).All data analyzed within this manuscript are publicly available. Skelly et al. mouse cardiac dataset can be downloaded from ArrayExpress (experiment E-MTAB-6173). FACS sorted cells sequenced with Smart-Seq2 from Tabula Muris are directly available for download from the figshare website: https://figshare.com/articles/Single-cell_RNA-seq_data_from_Smart-seq2_sequencing_of_FACS_sorted_cells/5715040. Single cells from mammary gland data of Tabula Muris Senis on the 10X Genomics Platform are also available for

April 2020

download from the figshare website: https://figshare.com/articles/Single-cell_RNA-seq_data_from_microfluidic_emulsion/5715025. The FANTOM5 CAGE expression data for human protein-coding genes in the 144 human primary cells are publicly available on FANTOM5 website (https://fantom.gsc.riken.jp/5/suppl/Ramilowski_et_al_2015/data/ExpressionGenes.txt). connectomeDB2020 is included in the NATMI repository (https://github.com/forrest-lab/NATMI). NCBI HomoloGene Database for ID conversion can be downloaded from https://ftp.ncbi.nih.gov/pub/HomoloGene/current/homologene.data

Field-specific reporting

Please select the one below that is the best fit for your research. If you are not sure, read the appropriate sections before making your selection.

☒ Life sciences ☐ Behavioural & social sciences ☐ Ecological, evolutionary & environmental sciences

For a reference copy of the document with all sections, see nature.com/documents/nr-reporting-summary-flat.pdf

Life sciences study design

All studies must disclose on these points even when the disclosure is negative.

Sample size	No sample size calculation was performed. All data used in this manuscript were taken from public resources and used to demonstrate the use of NATMI. We chose two organism-wide expression datasets (FANTOM5 and Tabula Muris) to cross-validate our observations. Tabula Muris Senis dataset has single-cell gene expression data at multiple time points which display the changes in cell populations and states. Therefore, it is sufficient to demonstrate the usage of delta network analysis in NATMI.
Data exclusions	No data were excluded.
Replication	All Attempts at replication were successful and can be performed independently.
Randomization	This is not relevant to our study because we only extract features from the datasets, there is no allocation procedure.
Blinding	Blinding was not possible because we only extract features from the datasets, there is no allocation procedure.

Reporting for specific materials, systems and methods

We require information from authors about some types of materials, experimental systems and methods used in many studies. Here, indicate whether each material, system or method listed is relevant to your study. If you are not sure if a list item applies to your research, read the appropriate section before selecting a response.

Materials & experimental systems

n/a	Involved in the study	
☒	☐	Antibodies
☒	☐	Eukaryotic cell lines
☒	☐	Palaeontology and archaeology
☒	☐	Animals and other organisms
☒	☐	Human research participants
☒	☐	Clinical data
☒	☐	Dual use research of concern

Methods

n/a	Involved in the study	
☒	☐	ChIP-seq
☒	☐	Flow cytometry
☒	☐	MRI-based neuroimaging
